# Characterisation of Regulatory T Cells in Nasal Associated Lymphoid Tissue in Children: Relationships with Pneumococcal Colonization

**DOI:** 10.1371/journal.ppat.1002175

**Published:** 2011-08-11

**Authors:** Qibo Zhang, Samuel C. Leong, Paul S. McNamara, Ayman Mubarak, Richard Malley, Adam Finn

**Affiliations:** 1 Department of Clinical Infection, Microbiology and Immunology, Institute of Infection and Global Health, University of Liverpool, United Kingdom; 2 Department of Otorhinolaryngology, Alder Hey Children's Hospital, Liverpool, United Kingdom; 3 Institute of Child Health, Alder Hey Children's Hospital, Liverpool, United Kingdom; 4 Children's Hospital and Harvard Medical School, Boston, Massachusetts, United States of America; 5 School of Cellular and Molecular Medicine, University of Bristol, Bristol, United Kingdom; National Institute of Allergy and Infectious Diseases, National Institutes of Health, United States of America

## Abstract

Regulatory T cells (Treg) diminish immune responses to microbial infection, which may contribute to preventing inflammation-related local tissue damage and autoimmunity but may also contribute to chronicity of infection. Nasopharyngeal carriage of pneumococcus is common in young children and can persist for long periods but it is unknown whether the presence of Treg in the nasopharynx contributes to this persistence. We have investigated the numbers and activities of Foxp3+Treg in adenoidal tissues and their association with pneumococcal carriage in children. Expression of Treg cell-related markers including Foxp3, CD25, CD39, CD127 and CLTA4 were analysed by flow-cytometry in adenoidal mononuclear cells (MNC) and PBMC from children. Unfractionated MNC or Treg-depleted MNC were stimulated with a pneumococcal whole cell antigen (WCA) and T cell proliferation measured. Cytokine production by MNC was measured using a cytometric bead array. Higher numbers of CD25^high^Foxp3^high^ Treg expressing higher CD39 and CTLA4 were found in adenoidal MNC than in PBMC. Children with pneumococcus positive nasopharyngeal cultures had higher proportions of Treg and expressed higher levels of CD39 and CTLA-4 than those who were culture negative (−). WCA induced adenoidal Treg proliferation which produce IL10 but not IL17, and CD4 T cell proliferation in Treg-depleted MNC was greater in pneumococcal culture positive than negative children. Significant numbers of Treg with an effector/memory phenotype which possess a potent inhibitory effect, exist in adenoidal tissue. The association of pneumococcal carriage with an increased frequency of adenoidal Treg suggests that Treg in nasal-associated lymphoid tissue (NALT) may contribute to the persistence of pneumococcus in children. Further studies to determine what component and mechanisms are involved in the promotion of Treg in NALT may lead to novel therapeutic or vaccination strategy against upper respiratory infection.

## Author Summary

*Streptococcus pneumoniae* (pneumococcus) is a bacterium that causes pneumonia, meningitis and blood poisoning. Colonization with pneumococcus is common in young children, which may be why they are prone to some common infections such as otitis media (ear infection) and pneumonia. As children age, most develop natural immunity to pneumococcus due to previous colonization. This immunity helps to prevent new infection and/or clear carriage of pneumococcus. However, persistence of carriage occurs in some children. The mechanisms for this are not clear. A good understanding of this phenomenon would help us to develop better ways to prevent pnemococcal infection. We have found that the immune tissues called adenoids (at the back of nose) in children contain some immune cells called “regulatory cells” that inhibit the naturally developed immunity to pnemococcus. While the presence and action of these cells is important to prevent self-tissue damage during infection (due to excessive immune response), they contribute to the persistence of pneumococcal carriage. We show evidence that these cells may develop from the action of some component of pneumococcus. Further studies are underway to determine what component and how it promotes these cells, which may lead to better vaccines to prevent pnemococcus and other similar infections.

## Introduction

Regulatory T cells (Treg) play a key role in the control of various aspects of the immune response including maintenance of immune tolerance and prevention of autoimmunity [1]. Progress has been made in recent years in the characterization of regulatory cells, including Foxp3+ Treg. Until recently, the expression of the transcription factor Foxp3+ on CD4 T cells was believed to indicate thymus-derived natural Treg. However, there is mounting evidence that Foxp3+ Treg also develop extrathymically, i.e. adaptive Treg [2]. Studies *in vitro* show conversion of naïve T CD4+CD25− T cells into Foxp3+ Treg through TCR ligation in the presence of TGF-β [3].

Up until now, intracellular expression of Foxp3 is still considered the most specific single marker of Treg, although a combination of phenotypic expression of CD4+CD25+CD127^low^ has also been established as a useful marker for natural Treg [4], [5]. Some phenotypic markers such as CD39 and CTLA-4 have been found to be associated with the activity of Treg [6]–[9]. In particular, CD39 expression on Treg has been found to be correlated with the inhibitory potency of Treg, and in humans it is considered to be a marker of effector/memory Tregs [10].

Recently, a growing number of studies suggest that Treg play an important role in the control of immunity to microbial pathogens including bacteria, viruses and parasites [11]. The repertoire of antigen specificities of Treg is considered to be broad, recognizing both self and non-self antigens. It has been suggested that Treg can be activated and expanded against a wide range of different pathogens *in vivo*. Such pathogen-specific Treg may prevent the infection-induced immunopathology but may prolong pathogen persistence by inhibiting protective immunity favoring chronicity of infections [12]. For example, Treg may contribute to the immunopathogenesis of chronic infections including Human Immunodeficiency virus (HIV), Hepatitis C virus (HCV) and Tuberculosis (TB) [13]–[15]. Mucosal Treg have been shown to be important in the modulation of gastrointestinal tract inflammation such as that related to Helicobacter pylori and HIV infection [12], [16]. However, data on mucosal Treg in the respiratory tract infections in humans are limited.

*Streptococcus pneumoniae* (pneumococcus) is a leading cause of bacterial pneumonia, meningitis and septicemia, and kills millions of people each year worldwide, especially children. Nasopharyngeal colonization with pneumococcus is common in young children, as are mucosal pneumococcal infections such as otitis media and pneumonia. By the age of three years, most children develop natural T- and B-cell specific immune responses to several pneumococcal protein antigens [17], [18] presumably due to previous colonization. These responses may protect against pneumococcal carriage either by preventing acquisition or hastening clearance or both and are induced in “nasal-associated lymphoid tissue”(NALT) [19], [20]. Nevertheless, pneumococcal nasopharyngeal carriage may be prolonged and may recur throughout life. Therefore we hypothesized that significant Treg activity in the nasopharynx may contribute to the persistence of pneumococcus and perhaps, recurrent infection in some cases, in children in the face of demonstrable mucosal specific immunity.

Establishment of chronic intracellular infections such as HIV and tuberculosis is associated with attenuated cell-mediated immunity [21], [22]. Pneumococcus is classically considered to be an extracellular bacterium against which antibody responses play a primary role in protection. However, recent studies in mice suggest that CD4+T cell-mediated immunity to pneumococcal protein antigens may play a major role in either preventing or reducing the duration of pneumococcal mucosal colonization [23]–[25]. Our own results also suggest that naturally developed CD4 T cell-mediated immunity to pneumolysin, an intracellular pneumococcal antigen released on autolysis, may be protective against pneumococcal carriage in children [18].

No previous data are available on the role of Treg in the regulation of CD4 T cell immunity to pneumococcus or other upper respiratory tract-colonising bacteria in humans. Understanding the regulation of naturally-acquired mucosal immunity should help inform the design of novel vaccination strategies against pneumococcal colonization and/or infection. In this study, we investigated both the association between numbers and activities of Treg in adenoids and nasopharyngeal carriage of pneumococcus in children, and the effects of Treg on CD4+ T cell responses induced by pneumococcus *in vitro*.

## Materials and Methods

### Patients and samples

Adenoidal tissues and peripheral blood samples were obtained from children (aged 3–6 years) undergoing adenoidectomy. Patients who received antibiotics or systemic steroids within 3 weeks of surgery or who had any known immunodeficiency were excluded from the study. A nasopharyngeal (NP) swab was taken on the day that the operation was performed which was stored and cultured for pneumococcus as described previously [18].

### Ethics statement

The study was approved by the local Research Ethics Committees (Liverpool Paediatric Research Ethics Committee and South Bristol local research ethics committee) and written informed consent was obtained from parents or carers in all cases.

### Cell separation and culture

Adenoidal mononuclear cells (MNC) and peripheral blood mononuclear cells (PBMC) were isolated by Ficoll gradient centrifugation (GE Healthcare). Depletion of CD25+ cells was performed using MACS magnetic microbeads separation (Miltenyi). To ensure purity, CD25+ cell-depleted cells were passed through a second column and purity was confirmed by CD4/CD25/CD127 and Foxp3 staining (both <1% positive in CD4 T cells by flow-cytometry). Adenoidal MNC, Treg-depleted MNC or PBMC were cultured in RPMI medium (containing penicillin, streptomycin and glutamine) with or without the addition of an ethanol-killed unencapsulated pneumococcal whole cell antigen (WCA) at 10^6^ or 10^7^ colony-forming unit (CFU) equivalents [26] for up to 7 days. The WCA was derived from strain Rx1AL-, a capsule- and autolysin-negative mutant described previously [26]. Briefly, the organism was grown to mid-log phase in Todd-Hewitt Broth supplemented with yeast extract, killed by the addition of 70% ethanol, after which the cell pellet was harvested and resuspended. A pneumolysin-negative WCA was made in the same fashion, using an Rx1AL- strain in which the pneumolysin gene was replaced by the Janus cassette, as previously described [27]. In some experiment, WCA was treated with proteinase K (200 ug/ml) for 1 hour at 37°C followed by heating (98°C, 30 min) before cell stimulation.

In some experiments, Treg were purified using magnetic microbeads (MACS, Miltenyi) following manufacturer's instructions. Briefly, CD4+ T cells were purified from adenoidal MNC using negative selection followed by positive selection of CD25+ cells. To ensure CD25^high^ T cell separation, the amount of anti-CD25 antibody-labelled microbead was titrated, and the optimal quantity was used. Also, to ensure purity, cells were passed through magnetic columns twice for each separation. Purity (>96%) of isolated Treg was confirmed by flow-cytometry following Foxp3 staining.

### Flow-cytometry

Multi-color flow-cytometry was performed to analyze the phenotypic expressions of different cell subsets. Cells were stained with fluorescence-labeled mouse anti-human antibodies to CD4, CD25 and CD127, CD39, CD69, CD45RO following standard procedures and analyzed on a FACSCalibur (BD Bioscience). Intracellular expressions of Foxp3 and CTLA-4 (CD152) were analysed by flow-cytometry following cell permeabilization (eBioscience).

Carboxyfluorescein diacetate 5,6 succinimidyl ester (CFSE)(Molecular Probes) was used to label adenoidal MNC or PBMC before cell culture, allowing for tracking of cell division after stimulation as described previously [18], [28]. Percentage of proliferative T cell subpopulations including Foxp3+ subsets was analyzed by CFSE and T cell marker staining followed by flow-cytometry.

### Measurement of cytokine production

Following cell culture and stimulation by antigens, cell culture supernatants were collected and analysed for production of IL2, IL4, IL5, IL10, IFNγ and TNFα using a cytometric bead array (BD Bioscience), and for IL17 using ELISA (R&D Systems) following manufacturers' instructions. Intracellular cytokine staining was performed in some experiments to determine the cellular sources of cytokine production after stimulation by WCA as described previously [29]. Briefly, adenoidal MNC were co-cultured with WCA for 6 hours and together with brefeldin A (eBioscience) in the last 4 hours. Cells were stained with fluorescence-labeled anti-human CD4, followed by fixation, permeabilization and staining with fluorescence-labeled Foxp3, anti-IL10 or anti-IL17 (BD Biosience), and were then analyzed by flow cytometry.

### Statistical analysis

Two-group comparisons were analyzed using Student's *t* test, and multiple group comparisons by ANOVA. Correlation was analysed by Pearson's correlation. Analysis was performed using SPSS software (SPSS version 16, SPSS Inc).

## Results

### Numbers of Treg and their expression of activation markers in adenoidal tissue

**Figure 1:**
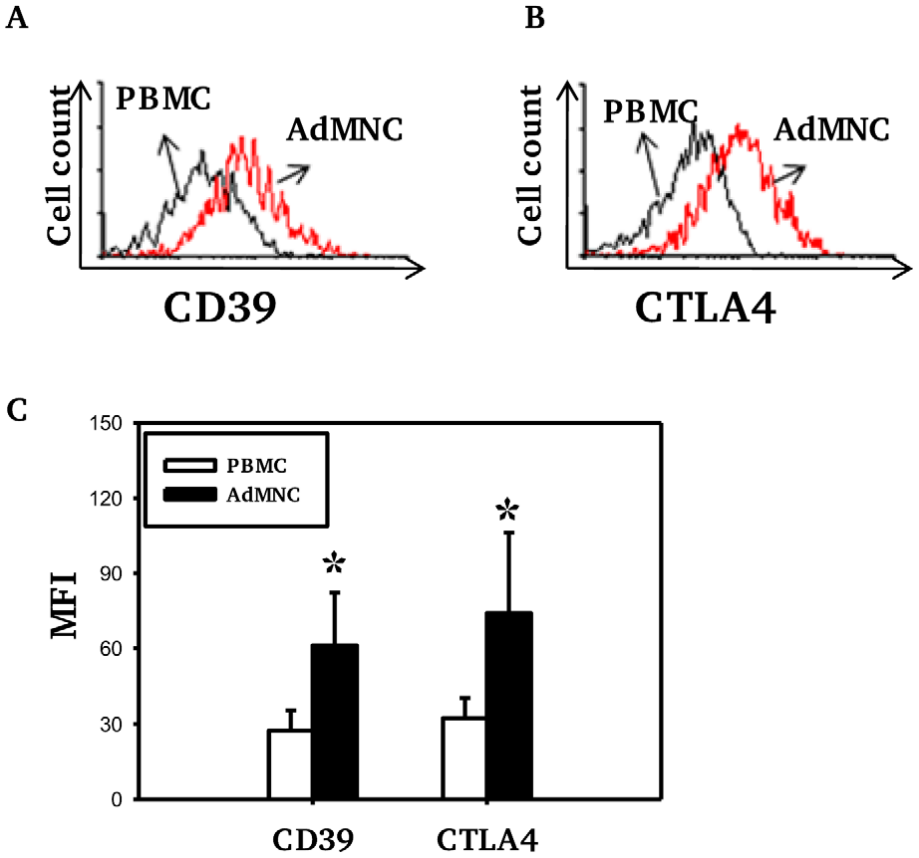
Expression of CD39 and CTLA4 by Foxp3+ Treg cells. Fluorescence histograms showing CD39 (A) and CTLA4 (B) expression by Foxp3+ Treg cells in PBMC (black) and adenoidal MNC (AdMNC) (red) from one representative of 20 replicate experiments summarized in panel C. (*p<0.01 compared to PBMC).

The numbers of Treg (as percentage of CD4 T cells) in both PBMC and adenoidal MNC were counted by staining for intracellular Foxp3 and/or CD4, CD25 and CD127. The use of CD4^+^CD25+ CD127^low^ as a phenotype for Treg correlated well with intracellular Foxp3 expression, both measured by flow-cytometry, in both PBMC and adenoidal MNC (r = 0.91 and 0.90 respectively, n = 12, p<0.01). The proportion of Foxp3+Treg in CD4+ T cells was higher in adenoidal MNC than PBMC (Table 1) and the percentages of adenoidal Treg expressing CD45RO or CD69 were also higher, in both cases, than in PBMC (both p<0.01, Table 1). Similarly, levels of CD39 and intracellular CTLA-4 expression were higher in adenoidal than in PBMC Treg (Fig. 1A–C).

**Table 1:** Proportion of Treg in CD4+T cells and percentages of Treg expressing CD45RO and CD69 in PBMC and adenoidal MNC.

	PBMC	Adenoidal MNC	n
% Treg in CD4 T cells	6.5(1.9)	11.9(3.9)*	20
% Treg expressing CD45RO	24.0(6.1)	52.0(7.1)**	20
% Treg expressing CD69	4.7(1.6)	67.2(5.8)**	20

Mean percentages (SD) are shown. (*p<0.05, **p<0.01 compared to PBMC).

**Figure 2:**
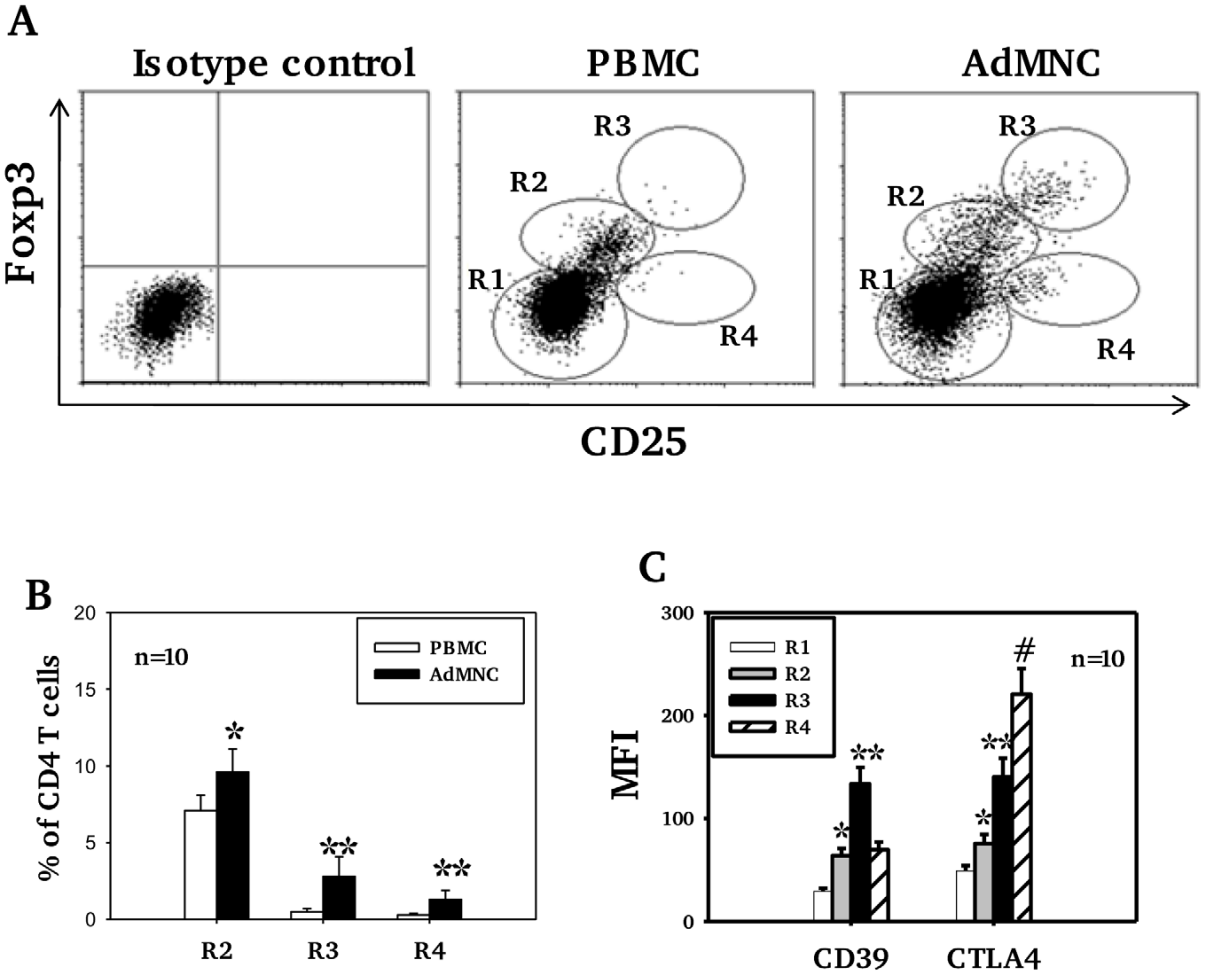
Expression of CD25, Foxp3, CD39 and CTLA4 by CD4 T cell subsets. In Fig A, R1 represents CD25^−^ Foxp3^−^ (non-Treg) CD4 T cells, defined as those with fluorescence comparable to isotype control staining. R2, R3 and R4 regions represent 3 distinctive cell populations shown in adenoidal MNC (ADMNC) with fluorescence above the top of fluorescence of isotype staining for Foxp3 and/or CD25. R3 represents the cell population with the highest fluorescence for both staining, defined as CD25^high^Foxp3^high^ Treg. R2 represents those cells with intermediate fluorescence (between R1 and R3) designated as CD25^intermediate^Foxp3^intermediate^ Treg. R4 represents the CD25^+^Foxp3^low^ cell population defined as activated CD4 T cells. One of 10 representative samples is shown in A. Fig B shows percentage of Foxp3+ Treg in PBMC and adenoidal MNC (*p<0.05, **p<0.01 compared to PBMC), and Fig C shows their expression of CD39 and CTLA4 (*p<0.01 compared to R1, **p<0.01 compared to R2 and R4, #p<0.01 compared to R3).

Higher levels of CD25 expression were observed in adenoidal Treg than in PBMC when paired samples from each individual were analyzed (data not shown, p<0.01). Co-staining for CD25 and Foxp3 showed significant numbers of Treg with a CD25^high^Foxp3^high^ phenotype in adenoidal MNC which were lacking in PBMC (Fig. 2A+B, region 3 (R3)). This phenotype of Treg exhibited higher levels of expression of both CD39 and CTLA-4 than CD25^intermediate^Foxp3^intermediate^ cells (Fig. 2C). In comparison with R3, R4 region (CD25^intermediate^Foxp3^low^) which represents activated CD4+ T cells, showed higher CTLA-4 but lower CD39 levels of expression (Fig. 2C).

### Adenoidal Treg in children with pneumococcal colonization

**Figure 3:**
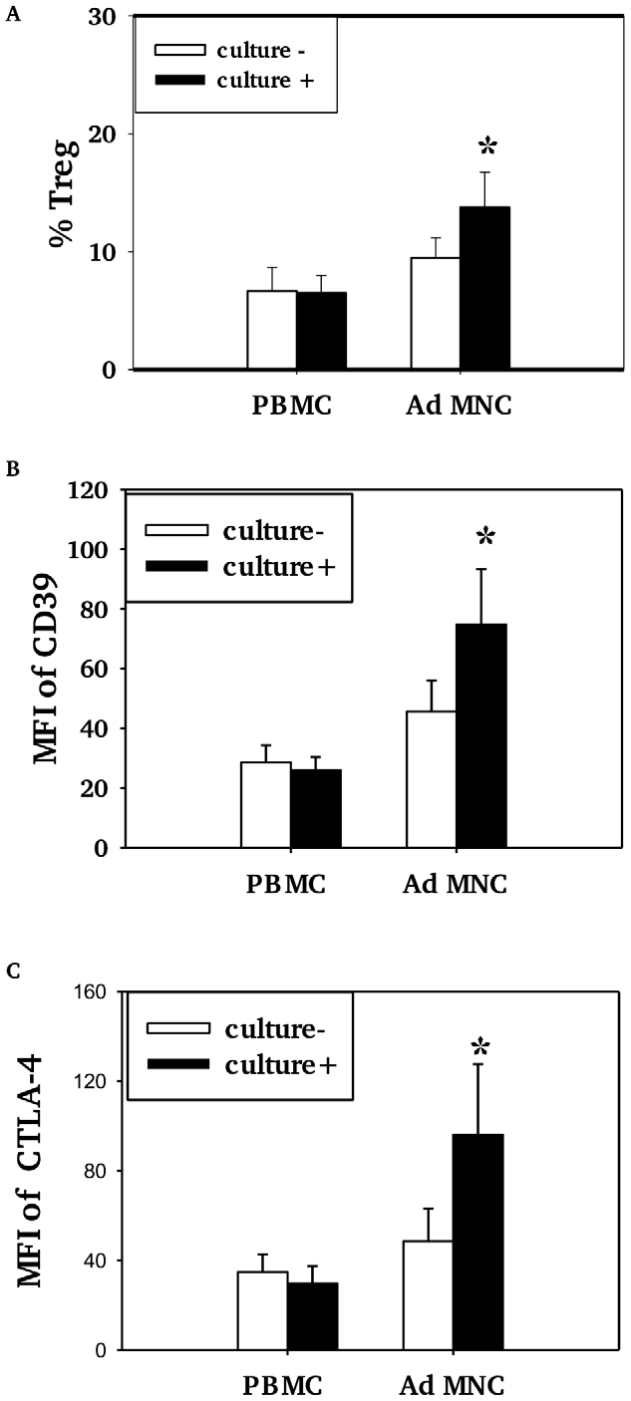
Association of pneumococcal carriage and Treg cells in adenoidal MNC. Percentages of CD4+Tcells which were Foxp3+ Treg (%Treg) (A) and their expression of CD39 (B) and intracellular CTLA4 (C) in PBMC and adenoidal MNC (AdMNC) in children who were culture positive (+) and negative (−) for pneumococcus. MFI – median fluorescence intensity. (*p<0.05 compared to culture negative children.) Culture+, n = 8; culture−, n = 12.

Children who had positive nasopharyngeal cultures for pneumococcus (+) had higher proportions of Treg (Foxp3+ and/or CD4/CD25/CD127^low^) among CD4+T cells in adenoidal MNC than those who were culture negative (−) (Fig. 3A, p<0.05) but no such difference in Treg numbers was seen in PBMC (Fig. 3A, p>0.05). The adenoidal Treg from culture-positive children also expressed higher levels of CD39 and intracellular CTLA-4, again with no such differences seen in PBMC (Fig. 3B and C).

### Pneumococcal WCA stimulation induces proliferation of Treg

**Figure 4:**
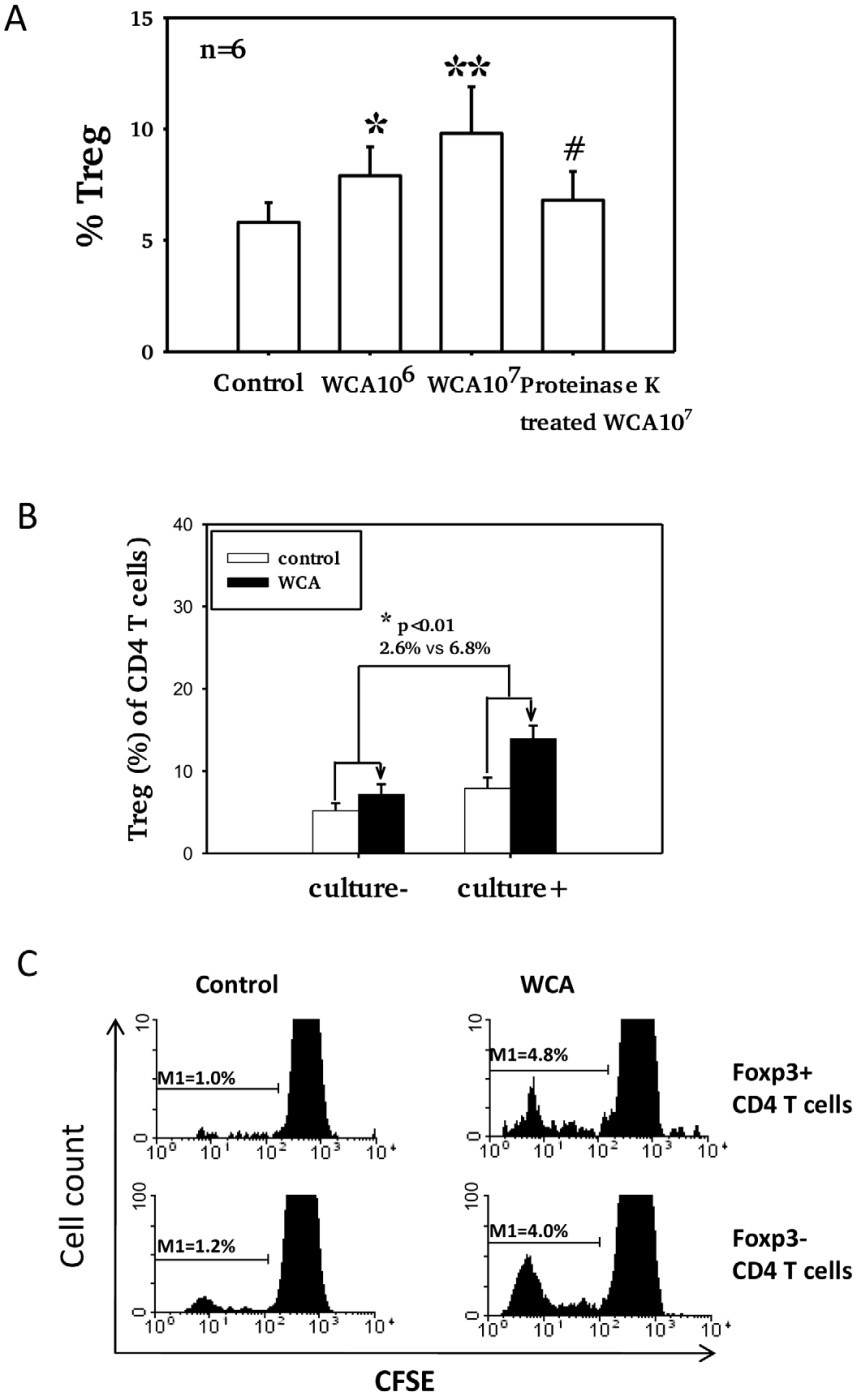
Pneumococcal WCA stimulation induces proliferation of Treg. A). Proportions of Foxp3+ Treg in adenoidal CD4+T cells after pneumococcal WCA stimulation at 10^6^ and 10^7^ (CFU equivalents) for 5 days. *p<0.0 1, **p<0.001 compared to control. #p<0.01 compared to WCA 10^7^ CFU equivalents. #p<0.01 compared to WCA 10^7^ CFU equivalents B). Mean % increase in Treg (of CD4+ T cells) after WCA stimulation (WCA 10^7^ CFU equivalents) in adenoidal MNC from pneumococcal culture+ (n = 6) and culture− children (n = 6)(p<0.01). C). CFSE staining shows proliferation of both Foxp3+ (Treg) and Foxp3− CD4+ T cells in adenoidal MNC following WCA stimulation. Result of one representative experiment of 6 replicates is shown.

Pneumococcal WCA stimulation of adenoidal MNC induces a dose-dependent increase in the numbers of Foxp3+ Treg compared to unstimulated control cells (Fig. 4A, p<0.01). Pre-treatment of pneumococcal WCA with proteinase K followed by heating significantly reduced this effect (Fig. 4A), suggesting that WCA-induced increase in Treg is protein-dependent. The mean % increase in the Treg (of CD4 T cells) after WCA stimulation is significantly higher in adenoidal MNC from pneumococcal culture+ children than from culture− children (Fig. 4B, p<0.01). A small increase was also observed in PBMC after stimulation with WCA (data not shown). Measurement of CD4+T cell proliferation using CFSE confirmed that stimulation with WCA induces both proliferation of Foxp3+ Treg and Foxp3− effector CD4+T cells in adenoidal MNC (Fig. 4C).

### Adenoidal Treg exhibit strong inhibition of CD4+ T cell responses to WCA

**Figure 5:**
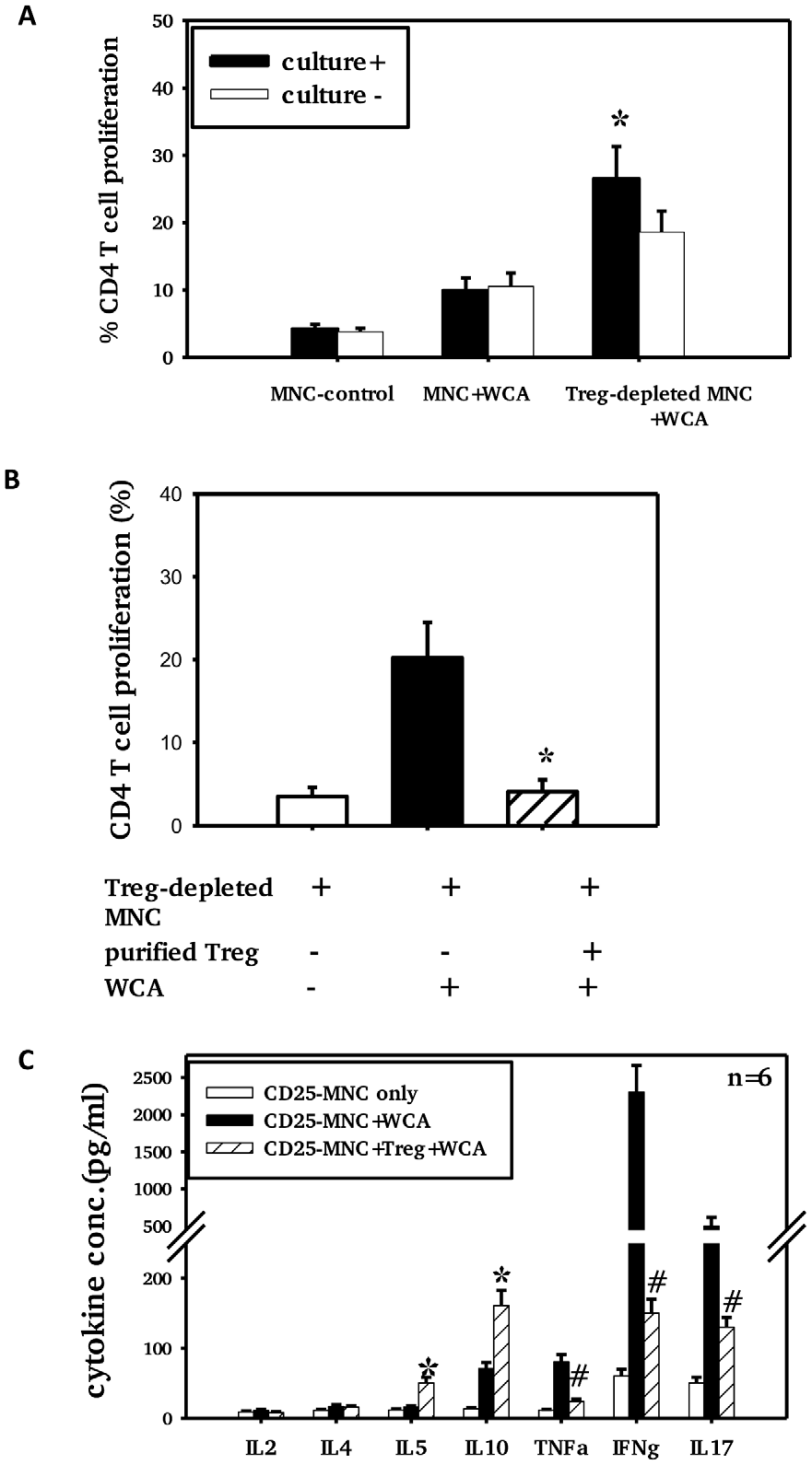
Adenoidal Treg exhibit strong inhibition of CD4+ T cell responses to WCA. CD4+ T cell proliferation (A+B) and cytokine production (C) in the presence or absence of Treg after stimulation with pneumococcal WCA. A). % CD4+ T cell proliferation in adenoidal MNC and Treg-depleted MNC from pneumococcal culture+ (n = 6) and culture− children (n = 6) (*p<0.01 compared to culture− group). B). CD4+ T cell proliferation induced by WCA in Treg-depleted MNC suppressed by the repletion of purified Treg (*p<0.01 compared to Treg-depleted MNC+WCA, n = 4. C). *p<0.01, #p<0.01 compared to Treg-depleted MNC+WCA.

To assess inhibition of T cell proliferation by adenoidal Treg, CD25+ cell-depleted adenoidal MNC were analyzed for CD4+ T cell proliferation following stimulation with pneumococcal WCA. WCA induced significantly higher CD4+ T cell proliferation in CD25+ cell-depleted MNC in those children who were pneumococcal culture positive than in those who were culture negative (Fig. 5a). The WCA-induced CD4+ T cell proliferation in CD25+cell-depleted MNC was suppressed when purified Treg (CD4+CD25+) were added back to the CD25+ cell-depleted MNC (Fig. 5B, p<0.01).

**Figure 6:**
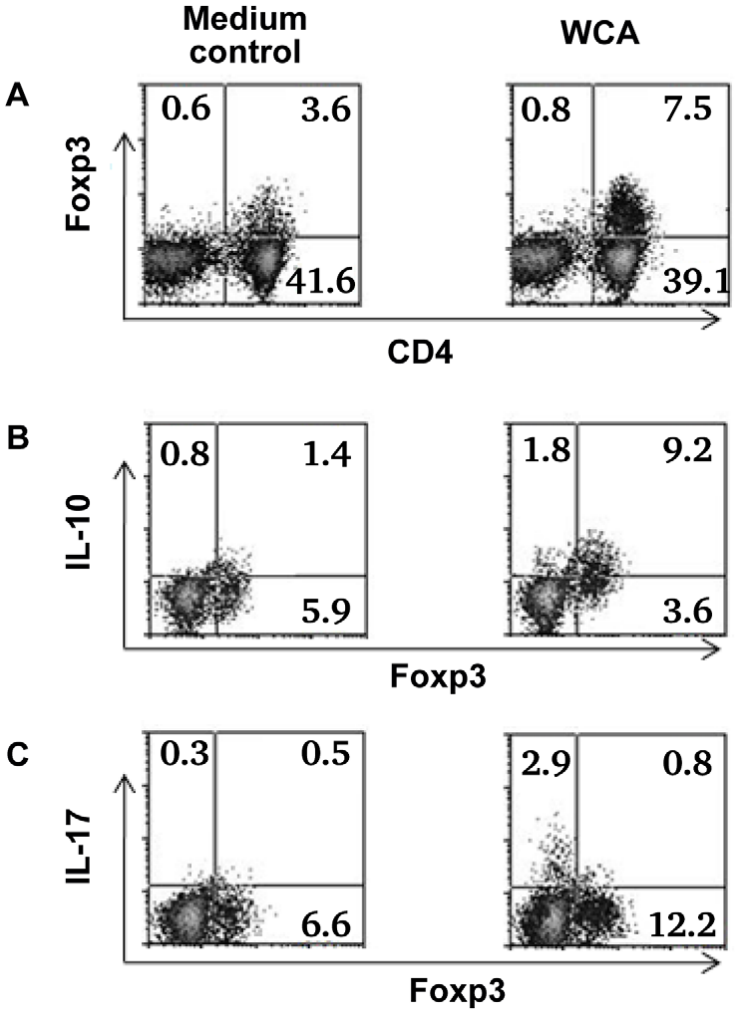
Cytokine expression by Foxp3+ Treg in adenoidal cells. Percentage of Foxp3+ Treg in adenoidal MNC after stimulation with WCA compared with unstimulated medium control (A). Intracellular cytokine staining shows IL-10 (B) and IL-17 (C) production by Foxp3+ Treg and Foxp3− CD4+ T cells after WCA stimulation compared with unstimulated control. Only CD4+ T cell gate is shown in B and C. Result of one representative experiment of four replicates is shown.

Depletion of Treg from adenoidal MNC resulted in a significant decrease in IL10 and IL5 but an increase in IL17, IFNγ and TNFα concentrations in the cell culture supernatant after WCA stimulation (data not shown). The increase in concentrations of IL17, IFNγ and TNFα in Treg-depleted adenoidal MNC following WCA stimulation was inhibited when purified Treg were reintroduced, which also restored the IL10 and IL5 production (Fig. 5C). Intracellular cytokine staining shows that Foxp3+ Treg in adenoidal CD4+ T cells secrete IL10 but not IL17 following stimulation by WCA (Fig. 6).

## Discussion

Recent studies have suggested that human Treg are functionally and phenotypically diverse. Foxp3+ Treg can be divided into naïve and effector/memory phenotypes according to the expression of CD45RA or RO, CD69 and CD25 [30]. Naïve Treg are characterized by their expression of CD45RA and low levels of Foxp3, and effector/memory Treg by expression of CD45RO and Foxp3^high^ [30], [31]. Although data in humans are lacking, animal studies suggest that CD45RA+Foxp3^low^ Treg are thymus-derived natural Treg [30], [31] which is supported by the finding that almost all Foxp3+ CD4 T cells found in human cord blood are CD45RA+Foxp3^low^ T cells [32], [33]. In this study, we found that the majority of Treg in PBMC in children express CD45RA (i.e. not expressing CD45RO), low levels of Foxp3, and do not express CD69 (Table 1). Thus these Treg are likely to be thymus-derived naïve Treg. In contrast, over 50% Treg in adenoidal tissues express CD45RO and CD69, and among them, a significant proportion expressing Foxp3^high^CD25^high^. Together with the high levels of CTLA-4 and CD39 expression (Fig. 1), these Foxp3^high^ CD25^high^Treg are likely to be of the effector/memory phenotype with potent suppressive properties [30], [34].

CD4+ T cells with the highest expression of CD25 were found to have the strongest suppressive activity, and thus CD25^high^ was used as a marker of Treg [34], However, in humans, levels of CD25 expression tend to show a continuous distribution and there is no consensus as to where the boundary lies between CD25^high^ and CD25^low to intermediate^ expression. In this study, with a combination of staining of Foxp3, CD4 and CD25, we demonstrate that Foxp3+ cells can be divided into two populations, Foxp3^high^CD25^high^ and Foxp3^intermediate^CD25^intermediate^ (Fig. 1A, region 3 and 2 respectively). Significant numbers of the Foxp3+ Treg in adenoids are of the former phenotype and express high levels of CD39, CTLA-4 and CD69. These results suggest that in human NALT there is a pool of effector/memory phenotype of Treg with potent suppressive function. These CD69+ Foxp3^high^CD25^high^ effector Treg differ phenotypically from the activated (non-Treg) CD4+ T cells which express intermediate to high levels of CD25 but low levels of Foxp3 and marked levels of CTLA-4 (Fig. 2A and C, region 4).

We demonstrate here that the proportion of Foxp3+ Treg, especially those of effector/memory phenotype, in adenoidal tissues is significantly higher than in peripheral blood. High numbers of Treg in NALT tissues could be induced by local colonization with microbes or antigens, as there is mounting evidence suggesting that the induction of Foxp3+ Treg occurs in peripheral tissues in humans. HIV, TB and Leishmania infections promote pathogen-specific Treg in the local inflammatory site including lymphoid tissues [35]–[38]. It has been postulated that the presence of microbial pathogens in peripheral tissues could lead to the accumulation of activated Treg (both natural and inducible) that help maintain host immune homeostasis [2], [35], [39].

In this study, we show that the proportion of adenoidal CD4+T cells that are Treg in adenoids, but not in peripheral blood, was significantly higher in children who were culture positive for pneumococcus in their nasopharynges than in those who were culture negative. This is the first report showing evidence of such an association for any extracellular pathogen in the human nasopharynx. This suggests that pneumococcal colonization in the nasopharynx may contribute to the induction and/or promotion of adaptive Treg in adenoids, and that these Treg may contribute to the delayed clearance or persistence of pneumococcal carriage in children. We also show that *in vitro* stimulation with a pneumococcal whole cell antigen (WCA) can induce an increase in the numbers of Treg in adenoidal MNC, and the increase is significantly higher in those children who are culture positive for pneumococcus. This would be consistent with the hypothesis that local colonization with pneumococcus promotes antigen-specific Treg *in vivo* in local lymphoid tissues adjacent to the site of colonization, which proliferate upon pneumococcal WCA stimulation.

To determine whether these Treg in adenoidal tissues are functional and induce antigen-specific inhibition of T cell responses, we compared the WCA-induced effector CD4 T cell proliferation in the presence or absence of Treg. We show that depletion of adenoidal Treg leads to marked increase in WCA-induced CD4+ T cell proliferation and proinflammatory cytokines including IL17, TNFα and IFNγ and replacement of the Treg abolished such effects. We also show that the increase in CD4+ T cell proliferation was significantly higher in children who were culture positive for pneumococcus than those who were culture negative. These results are consistent with the hypothesis that the adenoidal Treg are potent inhibitors and have antigen-specific inhibitory effects on CD4 T cell responses.

Recent data in mice suggest that Th17 cells which secrete IL17 may play a critical role in protection against nasopharyngeal carriage of pneumococcus through promoting neutrophil-mediated phagocytic killing [40]. It is now well recognized that Treg and Th17 are two T cell subsets with opposing actions and interplay in the regulation of inflammation and autoimmunity [41]. This is supported by our results here that adenoidal Treg secrete the inhibitory cytokine IL10 but not IL17 (Fig. 6). The results in this study suggest a potent inhibitory effect on Th17 cells by adenoidal Treg which would be consistent with a role of Treg in the persistence of pneumococcal carriage in children.

It is unclear what component of pneumococcus may contribute to the promotion of Treg in adenoids. Proteinase treatment of WCA reduced the inhibitory effect on WCA-induced CD4+ T cell proliferation, suggesting that pneumococcal protein(s) may contribute to the accumulation of adenoidal Treg. No difference was shown in the increased frequency of Treg induced by the wild-type WCA and an isogenic pneumolysin-negative WCA (data not shown), which suggests pneumolysin may not contribute significantly in this respect.

It has been reported previously that Treg numbers at the mucosal site, tonsil and lymph node, were highly increased in untreated patients with HIV infection, whereas in contrast, the Treg numbers in peripheral blood of these patients were not increased compared to healthy controls [12], [35], [42]. Taken together, these results support the hypothesis that chronic infection or persistent antigen promotes the expansion and activation of antigen-specific Treg in the local tissues [43].

In conclusion, significant numbers of Treg exist in adenoids of children with an effector/memory phenotype which possess potent inhibitory effect on CD4 T cell proliferation. The association of pneumococcal carriage in the nasopharynx with an increased frequency of Treg in adenoids suggests that local colonization with pneumococcus promotes pathogen-specific Treg of the effector/memory phenotype which may contribute to the delayed clearance or persistence of pneumococcus in children. Further studies to determine which pneumococcal components and what mechanisms are involved in the promotion of antigen-specific Treg in the nasopharynx may lead to novel therapeutic or vaccination strategies against upper respiratory colonization and/or infection.
